# Prevalence of arterial hypertension and risk factors among people with acquired immunodeficiency syndrome*

**DOI:** 10.1590/1518-8345.2684.3066

**Published:** 2018-10-25

**Authors:** Gilmara Holanda da Cunha, Maria Amanda Correia Lima, Marli Teresinha Gimeniz Galvão, Francisco Vagnaldo Fechine, Marina Soares Monteiro Fontenele, Larissa Rodrigues Siqueira

**Affiliations:** 1 Universidade Federal do Ceará, Departamento de Enfermagem, Fortaleza, CE, Brazil.; 2 Universidade Federal do Ceará, Centro de Pesquisa e Desenvolvimento de Medicamentos, Fortaleza, CE, Brazil.

**Keywords:** Acquired Immunodeficiency Syndrome, HIV, Hypertension, Nursing, Health Promotion, Antiretroviral Therapy, Highly Active

## Abstract

**Objectives:**

to verify the prevalence of arterial hypertension and its risk factors among people with acquired immunodeficiency syndrome under antiretroviral therapy.

**Method:**

cross-sectional study with 208 patients. Data collection was conducted through interviews using a form containing data on sociodemographic, clinical and epidemiological aspects, hypertension risk factors, blood pressure, weight, height, body mass index and abdominal circumference. Mean, standard deviation, odds ratio and confidence interval were calculated, t-test and Chi-square test were used, considering P < 0.05 as statistically significant. Hypertension associated variables were selected for logistic regression.

**Results:**

patients were male (70.7%), self-reported as mixed-race (68.2%), had schooling between 9 and 12 years of study (46.6%), had no children (47.6%), were single (44.2%), in the sexual exposure category (72.1%) and heterosexual (60.6%). The prevalence of people with acquired immunodeficiency syndrome and arterial hypertension was 17.3%. Logistic regression confirmed the influence of age greater than 45 years, family history of hypertension, being overweight and antiretroviral therapy for more than 36 months for hypertension to occur.

**Conclusion:**

the prevalence of hypertension was 17.3%. Patients with acquired immunodeficiency syndrome and hypertension were older than 45 years, had family history of hypertension, were overweight and under antiretroviral therapy for more than 36 months.

## Introduction

Acquired immunodeficiency syndrome (aids) represents one of the greatest contemporary health problems, due to its pandemic character and severity, being a challenge due to the nonexistence of an effective treatment that results in cure, in addition to socioeconomic barriers that interfere with the adherence to the treatment regimen^1^. Antiretroviral therapy (ART) is the only treatment available that provides the increased survival and decreases mortality, characterizing the disease as chronic. Thus, the treatment focus has shifted from the disease itself and immunodeficiency-related opportunistic infections to long-term problems caused by the effects of the human immunodeficiency virus (HIV) and ART, which includes toxicity, drug interactions or resistance to these drugs^2^.

Moreover, an increase in the frequency of cardiovascular disease in people with aids has been observed, especially hypertension, which is characterized by systolic blood pressure greater than or equal to 140 mmHg and diastolic blood pressure greater than or equal to 90 mmHg^3^ ^-^ ^4^. However, it is not known whether this is related to increases in the patients’ survival rate, whom now reach older ages; if it is related to the HIV infection itself; if it can be assigned to ART as a result of adverse drug events; or, if all of these factors contribute synergistically to the occurrence of cardiovascular diseases^2^ ^-^ ^5^ ^).^

Studies also show that many people with aids have unhealthy lifestyles regarding their feeding, exercise, alcohol consumption and smoking habits, in addition to traditional hypertension risk factors, which include advanced age, male gender, African ascent, high body mass index (BMI) and high cholesterol^3^ ^,^ ^6^. Thus, efforts to reduce cardiovascular risk in patients using ART should focus on prevention and control of hypertension, because this is a common, known and modifiable predictive factor^3^ ^-^ ^4^.

Data on prevalence of hypertension among people with aids are variable. Although some authors report higher prevalence of hypertension in this group^7^ ^-^ ^8^, when compared to uninfected individuals, other studies present similar prevalence of hypertension among men and women with aids and individuals uninfected by HIV^9^ ^-^ ^10^.

There are justifications for a study on the risk factors for hypertension and its prevalence among aids patients. The first is that the prevention of cardiovascular diseases is important for these patients due to predisposition, the HIV infection, the use of ART and aging due to the increase in the survival rate^4^ ^,^ ^11^. In addition, detection, treatment and control of hypertension are fundamental to reduce cardiovascular diseases, since they increase the number of hospitalizations and lead to high medical and socioeconomic costs^12^.

The efforts of health professionals, scientific community and government agencies are essential to treat and control hypertension. Such studies are important so health care professionals can perform preventive and treatment measures of cardiovascular diseases, given that health promotion practices are critical for these patients, whom require specialized care to maintain their quality of life.

Given this context, an interdisciplinary approach is required in the follow-up of people living with HIV/aids, mainly due to changes in lifestyle and frequent monitoring. Among health professionals, nurses have a strategic role and provide care to aids patients in different health areas. The nurse must comprehend the disorder, improve routine practices, adopt preventive measures to avoid the accidental exposure to HIV, and acquire knowledge of clinical treatment in its different aspects^13^.

In this perspective, considering the increase in the survival rate of people with aids by the implementation of ART, the known actions of HIV on the body, the adverse events of ART and the increase of cardiovascular diseases in these individuals showed by the studies cited - having hypertension as one of its primary precursors-, the general objective of this study was to verify the prevalence of hypertension and its risk factors among people with aids under antiretroviral therapy. This research can direct health practices of nurses and other professionals who provide care for these patients.

## Method

This is a cross-sectional, descriptive and quantitative study, developed in the infectious diseases outpatient clinic of the University Hospital Walter Cantídio of the Universidade Federal do Ceará (UFC), in Fortaleza, Ceará, Brazil, from August 2015 to August 2017. The study population was constituted by aids patients whom were treated in the clinic.

A sample was scaled to estimate the prevalence of aids patients served in the outpatient clinic and who had hypertension and its risk factors, with 95% confidence that the estimation error does not exceed 5%, considering that such prevalence is unknown in the population, being stipulated in 50% (presumed prevalence) - as it provides the largest sample size -, and that there were 450 patients under ART being served during the study period. For such, the following expression was used: n = z^2^×*p*× (1 -*p*) ×*N*/ ε^2^× (*N*- 1) + z^2^×*p*× (1 -*p*). In this formula, *z^2^* is equal to the value of the *z* statistic (1.96) for the adopted degree of confidence (95%) and *p*, *N* and ε correspond to the assumed prevalence (0.50), to the population (450) and to the tolerable error (0.05), respectively. Thus, a sample of 208 patients was calculated.

Inclusion criteria were people with aids of both genders, aged 18 years or older, who were under ART for at least three months and who were in outpatient follow-up. Exclusion criteria were pregnancy, mental illness, persons deprived of their liberty, people living in collective shelters or any other condition capable of interfering in an individual’s participation in the research.

The sampling strategies adopted were non-probability and convenience. Patients were invited to participate in the study when they attended to routine consultations in the outpatient clinic. Those who agreed to participate in the research signed an informed consent form, and were interviewed for approximately 40 minutes in a private environment.

A form divided into two parts was used: I. sociodemographic, epidemiological and clinical variables (age, sex, skin color, schooling, marital status, number of children, religion, occupational situation, monthly family income, category of exposure, sexual orientation, presence of lipodystrophy, use of anti-retroviral drugs, CD4+ T lymphocytes count, viral load, time of infection, time of use of ART); II. variables related to hypertension and its risk factors (salt consumption, use of salt shaker on the table, use of alcohol, smoking, exercising, personal and family background of hypertension, daily consumption of fruits, vegetables, fried and fatty foods, hypertension diagnosis and antihypertensive drugs used), blood pressure measurement (normal: ≤ 120/80 mmHg; hypertension: ≥ 140/90 mmHg), weight, height, body mass index (normal: < 25 kg/m^2^; overweight: ≥ 25 kg/m^2^;obesity: ≥ 30 kg/m^2^) and waist circumference (normality in men and women, < 94 and < 80 cm, respectively).

Part I of the form had already been validated in previous studies^14^ ^-^ ^15^, the data on hypertension and its risk factors were added to it. Prior to data collection, the complete form was applied to 20 aids patients whom were not part of the sample. The study researchers were trained to apply the form, considering subjective and objective data, using standard operating procedures for measuring blood pressure, weight, height, BMI and waist circumference and to set the normality parameters of the findings^16^.

The mean and standard deviation were calculated in the statistical analysis. For comparisons between hypertensive and normotensive subjects, the t-test was used for unpaired variables. A P < 0.05 was considered a statistically significant value. Absolute and relative frequencies were determined. The association of sociodemographic and clinical factors and the occurrence of hypertension, which is the primary outcome, were evaluated by the Chi-square test, considering P < 0.05 as statistically significant. The strength of such association also was evaluated by determining the odds ratio and its 95% confidence interval. Explanatory variables associated with hypertension at 20% significance level (P < 0.2) were selected to be part of the logistic regression model, identifying those that, independently, were factors associated with hypertension. For such, the stepwise backward method was used. The criterion for removing variables from the model was defined by the Wald test. This analysis was used to calculate the adjusted odds ratio, accuracy (95% confidence interval) and significance (Wald’s test) of the estimate. The Statistical Package for the Social Sciences (SPSS) software 20.0 version was used for the statistical procedures.

The project was approved by the Research Ethics Committee of UFC on March 12, 2015, under opinion nº 983.195. All participants signed the informed consent form. The participants’ privacy was maintained and the research data used only for scientific purposes. This study also considered the STROBE Statement guidelines.

## Results

Of the 208 people with aids evaluated, most were male (70.7%), self-reported as mixed-race (68.2%), schooling from 9 to 12 years of study (46.6%), single (44.2%) or married (41.1%) and had no children (47.6%). Most reported being Catholic (66.4%), were employed at the time of the study (55.3%) and had monthly family income greater than three minimum wages (26.4%). Most were in the sexual exposure category (72.1%), straight (60.6%) and 88 had lipodystrophy (42.3%). Data presented in Table 1.

**Table 1:** Sociodemographic and epidemiological characterization of people with acquired immunodeficiency syndrome (n = 208). Fortaleza, Ceará, Brazil, 2015-2017

Sociodemographic and epidemiological variables	N	%
Sex		
Male	147	70.7
Female	61	29.3
Skin color		
White	48	23.1
Black	18	8.7
Mixed-race	142	68.2
Schooling (years of study)		
≤ 8 years (illiterate or some elementary/middle school)	60	28.9
9 - 12 years (Elementary or High School)	97	46.6
≥ 13 years (higher education)	51	24.5
Marital status		
Single	92	44.2
Married	86	41.4
Divorced/widowed	30	14.4
Number of children		
None	99	47.6
1 - 2	67	32.2
≥ 3	42	20.2
Religion		
Catholic	138	66.4
Evangelical	36	17.3
Others (no religion, Spiritualist, Umbanda)	34	16.3
Occupational situation		
Employed	115	55.3
Unemployed	54	25.9
Retired/temporary retirement	39	18.8
Monthly family income in number of minimum wages*		
< 1	47	22.6
1 - 2	70	33.7
2 - 3	36	17.3
> 3	55	26.4
Exposure category		
Sexual	150	72.1%
Blood/transfusion	6	2.9%
Puncture or cutting accident	1	0.5%
Unknown	51	24.5%
Sexual orientation		
Heterosexual	126	60.6%
Homosexual	64	30.8%
Bisexual	18	8.6%
Lipodystrophy		
Yes	88	42.3%
No	120	57.7%

*Current minimum wage in Brazil in the study period - 2015: R$ 788.00; 2016: R$ 880.00; 2017: R$ 937.00

Among the antiretroviral drugs used were: lamivudine (195; 94%), tenofovir (125; 60.1%), efavirenz (116; 55.8%), zidovudine (93; 44.7%), atazanavir (42; 20.2%), lopinavir (27; 13%), nevirapine (11; 5.3%) and raltegravir (6; 2.9%). Regarding the values of HIV-related laboratory tests, considering the 208 patients, was found: CD4+ T lymphocytes (cells/mm^3^) (mean ± standard deviation: 599.144 ± 377.960; minimum value: 29; maximum value: 3.179) and viral load (copies/ml) (mean ± standard deviation: 18.027.086 ± 104.133.463; minimum value: 0; maximum value: 1.058.662).

Most people with aids reported moderate salt consumption (56.7%) and 26 patients (12.5%) used a salt shaker on the table during meals. Regarding food, most patients reported daily consumption of fruits (92.3%), vegetables (91.3%), fried and fatty foods (78.8%). A considerable number of patients used alcohol (40.4%), 54 (26%) had stopped smoking and 19.7% were smokers. Most did not practice physical exercises (61.5%), 141 (67.8%) had family history of hypertension, and the main personal history was diabetes (6.7%) (Table 2).

**Table 2:** Risk factors for hypertension presented by people with acquired immunodeficiency syndrome (n = 208). Fortaleza, Ceará, Brazil, 2015-2017

Risk factors for arterial hypertension	N	%
Salt consumption		
High	28	13.5
Moderate	118	56.7
Low	62	29.8
Presence of salt shaker on the table		
Yes	26	12.5
No	182	87.5
Consumption of alcoholic beverages		
Yes	84	40.4
No	124	59.6
Smoking habit		
Never smoked	113	54.3
Stopped smoking	54	26.0
Smoker	41	19.7
Performance of physical activities		
Yes	80	38.5
No	128	61.5
Family history of hypertension		
Yes	141	67.8
No	67	32.2
Personal background		
Diabetes	14	6.7
Cerebrovascular accident	5	2.4
Myocardial Infarction	4	1.9
Angina	1	0.5
Daily consumption of fruits		
Yes	192	92.3
No	16	7.7
Daily consumption of vegetables		
Yes	190	91.3
No	18	8.7
Daily consumption of fried and fatty foods		
Yes	164	78.8
No	44	21.2

In the sample of 208 patients, 36 had hypertension, with 17.3% prevalence (95% confidence interval: 12.1 - 22.4%). The antihypertensive drugs used were: losartan (18; 50%), hydrochlorothiazide (11; 30.6%), enalapril (8; 22.2%), propranolol (4; 11.1%), atenolol (4; 11.1%), amlodipine (3; 8.3%), captopril (2; 5.6%), carvedilol (1; 2.8%), chlortalidone (1; 2.8%), furosemide (1; 2.8%) and metoprolol (1; 2.8%). The association between sex and hypertension was evaluated by the Chi-square test. The t-test was used for unpaired data to compare the two strata in relation to other variables. It was found that people with aids and hypertension had higher mean age (P < 0.001), greater waist circumference (P < 0.001), longer time of infection (P = 0.005) and longer time of use of ART (P = 0.002) (Table 3).

**Table 3:** Sociodemographic and clinical characteristics of people with acquired immunodeficiency syndromed stratified according to the presence of arterial hypertension (n = 208). Fortaleza, Ceará, Brazil, 2015-2017

Characteristics	Systemic arterial hypertension		Significance
	Present	Absent	
Age (years, mean ± SD*)	48.8 ± 12.0	39.7 ± 10.6	P < 0.001
Sex, n (%)			
Male	27 (75.0%)	120 (69.7%)	P = 0.531
Female	9 (25.0%)	52 (30.2%)	
Body mass index (kg^†^/m^2‡^, mean ± SD*)	27.0 ± 4.3	25.4 ± 6.9	P = 0.193
Waist circumference (cm^§^, mean ± SD*)	96.2 ± 9.9	88.2 ± 11.2	P < 0.001
Time of HIV infection (years, mean ± SD*)	8.6 ± 4.0	6.3 ± 4.5	P = 0.005
Time of use of ART^||^ (months, mean ± SD*)	92.7 ± 44.6	62.6 ± 54.0	P = 0.002
CD4+ T lymphocytes count (cells/mm^3¶^, mean ± SD*)	612.4 ± 281.6	596.3 ± 395.8	P = 0.817

*SD: standard deviation; †kg: kilogram; ‡m^2^: square meter; §cm: centimeter; ||ART: antiretroviral therapy; ¶mm^3^: cubic milimeters

The association between the risk factors for hypertension and the occurrence of hypertension was evaluated by the Chi-square test, and by determining the odds ratio and its respective 95% confidence interval (95% CI). Table 4 shows the data expressed as number of cases (n) and percentage (%). It was observed that people with aids had higher chances of presenting hypertension when the age was greater than 45 years (P = 0.003), had family history of hypertension (P = 0.003), were overweight (P = 0.024), had increased waist circumference (P = 0.013) and time of use of ART greater than 36 months (P < 0.001) (Table 4).

**Table 4:** Associated factors with hypertension in people with acquired immunodeficiency syndrome in use of antiretroviral therapy according to the presence (n = 36) or absence (n = 172) of arterial hypertension. Fortaleza, Ceará, Brazil, 2015-2017

Risk factors for arterial hypertension	Hypertension				OR*	95% CI^†^	Significance (Chi-square test)
	Present		Absent				
	n	%	n	%			
Age							
> 45 years	20	55.5	51	29.6	2.97	1.42 - 6.18	P = 0.003
≤ 45 years	16	44.4	121	70.3	1		
Sex							
Male	27	75.0	120	69.7	1.30	0.57 - 2.96	P = 0.531
Female	9	25.0	52	30.2	1		
Family history of hypertension							
Yes	32	88.8	110	63.9	4.51	1.52 - 13.4	P = 0.003
No	4	11.1	62	36.0	1		
Smoking							
Yes	12	33.3	83	48.2	0.54	0.25 - 1.14	P = 0.102
No	24	66.6	89	51.7	1		
Consumption of alcohol							
Yes	15	41.6	69	40.1	1.07	0.51 - 2.21	P = 0.863
No	21	58.3	103	59.8	1		
Physical activity							
Yes	13	36.1	67	38.9	0.89	0.42 - 1.87	P = 0.750
No	23	63.8	105	61.0	1		
Overweight (BMI^‡^ ≥ 25)							
Yes	24	66.6	79	45.9	2.35	1.11 - 5.01	P = 0.024
No	12	33.3	93	54.0	1		
Obesity (BMI^‡^ ≥ 30)							
Yes	8	22.2	22	12.7	1.95	0.79 - 4.81	P = 0.143
No	28	77.7	150	87.2	1		
Waist circumference							
Increased	14	38.8	34	19.7	2.58	1.20 - 5.57	P = 0.013
Normal	22	61.1	138	80.2	1		
CD4+ T lymphocytes count							
< 350 cells/mm^3§^	7	19.4	41	23.8	0.77	0.31 - 1.89	P = 0.569
≥ 350 cells/mm^3§^	29	80.5	131	76.1	1		
Time of diagnosis of HIV^||^ infection							
> 3 years	34	94.4	142	82.5	3.59	0.82 - 15.77	P = 0.072
≤ 3 years	2	5.5	30	17.4	1		
Time of antiretroviral therapy							
> 36 months	31	86.1	91	52.9	5.52	2.05 - 14.86	P < 0.001
≤ 36 months	5	13.8	81	47.0	1		

*OR: odds ratio; †CI: confidence interval; ‡BMI: body mass index; §mm3: cubic millimeters; ||HIV: human immunodeficiency virus

Logistic regression analysis was used to determine the adjusted odds ratio, as well as the accuracy (95% confidence interval) and significance (Wald’s test) of the estimate. The variables that integrated the logistic regression model (P < 0.2) were: age, family history of hypertension, smoking, overweight, obesity, waist circumference, time of HIV diagnosis and time of use of ART. The results of the analyses showed that in the considered sample, the risk of hypertension increased with the following variables: age greater than 45 years (P = 0.01), family history of hypertension (P = 0.005), being overweight (P = 0.019) and time of use of ART (P = 0.002) (Table 5).

**Table 5:** Determination of the factors associated with hypertension in people with acquired immune deficiency syndrome under antiretroviral therapy, after control of the possible confounding variables (n = 36). Fortaleza, Ceará, Brazil, 2015-2017

Factor	Crude odds ratio	Adjusted odds ratio	95% CI*	Significance (Wald’s test)
Age				
> 45 years	2.97	2.95	1.30 - 6.70	P = 0.010
≤ 45 years	1	1		
Family history of hypertension				
Yes	4.51	5.12	1.64 - 15.98	P = 0.005
No	1	1		
Overweight (BMI^†^ ≥ 25)				
Yes	2.35	2.74	1.18 - 6.36	P = 0.019
No	1	1		
Time of antiretroviral therapy				
> 36 months	5.52	4.99	1.77 - 14.05	P = 0.002
≤ 36 months	1	1		

*CI: confidence interval; ^†^BMI: body mass index

## Discussion

Most of the sample of this study were men, corroborating with other studies, showing that HIV is still affecting more men than women^17^ ^-^ ^18^. People of darker skin color were highlighted in the analyses, and in this regard, a study conducted in the United States also pointed that the absolute number of Caucasians with an aids diagnosis is much lower when compared to African Americans; however, there is a tendency in the number of people with aids increasing among Caucasian individuals when compared to African Americans^19^.

The patients’ schooling was relatively high, similar to the findings of other studies^20^ ^-^ ^21^. People with higher education level may have more access to relevant health information, presenting a broader perception on cardiovascular risk factors and the need to maintain a healthy lifestyle^22^. Singles were the majority of patients and, given this, our study showed that single people are more likely to have multiple partners, thus becoming more vulnerable to HIV^23^. However, the increase in the number of HIV infection cases among people in stable relationships must be highlighted; this derives from the lack of negotiation about condom use, especially by females^24^.

Participants whom did not had children were the majority. On this aspect, a study showed that the care demands of several children, especially if they are young, can lead to problems in the treatment routine, due to competing needs from the children’s routine^25^. Regarding religion, most participants reported to be Catholic. Regardless of belief, this study found that religion helps people with aids in adhering to the ART and in the fight against the disease, but it must be noted that mistakes can happen and some patients may start assigning the treatment and cure of aids to religion, not properly adhering to the ART and other health guidelines^26^.

Most patients were employed during the study period. Having a steady job can help people with aids to replace their identity as patients, since those who work report significant improvements in their quality of life when compared to those who are unemployed; however, usually the main cause of cessation of work is the stigma resulting from the disease^27^. Sexual exposure and heterosexuality were relevant in the analyses, contrasting the beginnings of the epidemic, when most people affected were homosexuals, users of intravenous drugs and people who underwent blood transfusions^1^. A considerable number of patients had lipodystrophy. Considering this information, a research showed that adherence to ART tends to decrease over time after the lipodystrophy diagnosis^28^.

The antiretroviral drugs used the most were lamivudine, tenofovir efavirenz and zidovudine. Considering these drugs, nucleoside analogue reverse transcriptase inhibitors such as lamivudine, zidovudine and tenofovir, may cause mitochondrial and liver toxicity, lipoatrophy, anemia, myopathy, peripheral neuropathy and pancreatitis. On the other hand, non-nucleoside analogue reverse transcriptase inhibitors like efavirenz, may elevate liver enzymes, cause dyslipidemia, exanthema and Stevens-Johnson syndrome^29^.

Regarding the risk factors for hypertension, most aids patients presented moderate salt consumption and some used salt shaker on the table during meals. Low salt consumption and not using a salt shaker on the table are hypertension prevention strategies that must be adopted by everyone^30^. Many patients ate fried and fatty foods, which can lead to obesity and cardiovascular diseases^31^. Some participants used alcohol and smoked, which is also related to the development of cardiovascular diseases^32^ ^-^ ^33^. Most had family history of hypertension, diabetes mellitus and did not practice physical exercises.

In this study, the prevalence of hypertension among people with aids was 17.3%. However, data on prevalence of hypertension among this type of patient are variable. A survey found 19.3% prevalence of people with aids before starting ART, but after 12 months of ART initiation, 31% of the patients were hypertensive^34^. In another study, the prevalence ranged from 4.7% and 54.4% in high-income countries and from 8.7% to 45.9% in middle-income countries^6^. Another research found prevalence of hypertension in 38% of people with aids under ART, and 19% in people whom were not under such therapy^4^.

In this study, the antihypertensive drugs most frequently used by people with aids were losartan, hydrochlorothiazide and enalapril.It was found that people with aids and hypertension had higher mean age, greater circumference waist, longer time of infection and longer time of use of ART.

Patients with aids had higher chances of presenting hypertension when the age was greater than 45 years, had family history of hypertension, were overweight, had increased waist circumference and time of use of ART greater than 36 months. Logistic regression analysis showed that the risk of hypertension increased according to age greater than 45 years, family history of hypertension, being overweight and time of use of ART.

We can assume that the incidence of cardiovascular diseases in people with aids increases due to the profile of high risk factors and increased the survival rate of these patients. Therefore, the estimation of cardiovascular risk and the management of these risk factors among individuals with aids must be part of the regular treatment approach^35^.

Considering the limitations of the study, one of them was not thoroughly verifying what types of antiretroviral drugs were more associated with hypertension, because all aids patients used a combination of distinct classes of antiretroviral drugs. Another relevant aspect would be the inclusion of a control group of patients with aids whom were not under ART. This was not possible due to recent guidelines for the treatment of people with aids, which advocate the use of ART as soon as possible after the positive diagnosis of anti-HIV serology, as a measure to decrease the morbidity and mortality among these patients.

## Conclusion

This study concludes that the prevalence of people with aids and hypertension was 17.3%. In the studied sample, patients with aids and hypertension were older than 45 years, had family history of hypertension, were overweight (BMI ≥ 25), had increased waist circumference and used ART for more than 36 months. Finally, the logistic regression analysis confirmed the influence of age greater than 45 years, family history of hypertension, being overweight (BMI ≥ 25) and use of ART for more than 36 months in the process of hypertension of patients with aids assessed in this research.

We emphasize the importance of this study, given that ART reduced the morbidity and mortality of people with aids, providing greater survival rates. Therefore, the analysis of diseases that affect the general population is important among people with aids, seeking to provide a better quality of life for these individuals.
